# Supplementary material for: Netcombin: An algorithm for constructing optimal phylogenetic network from rooted triplets
Source: PLoS One. 2020 Sep 18;15(9):e0227842. doi: 10.1371/journal.pone.0227842 (PMC7500971; doi:10.1371/journal.pone.0227842)
Supplement: S4 File — (ZIP) [file pone.0227842.s004.zip › SequenceData-Triplet/sequenceData-10-20-30-0-Triplet/length 200, numseq 40, rate 10^-7/10 seq.pdf]

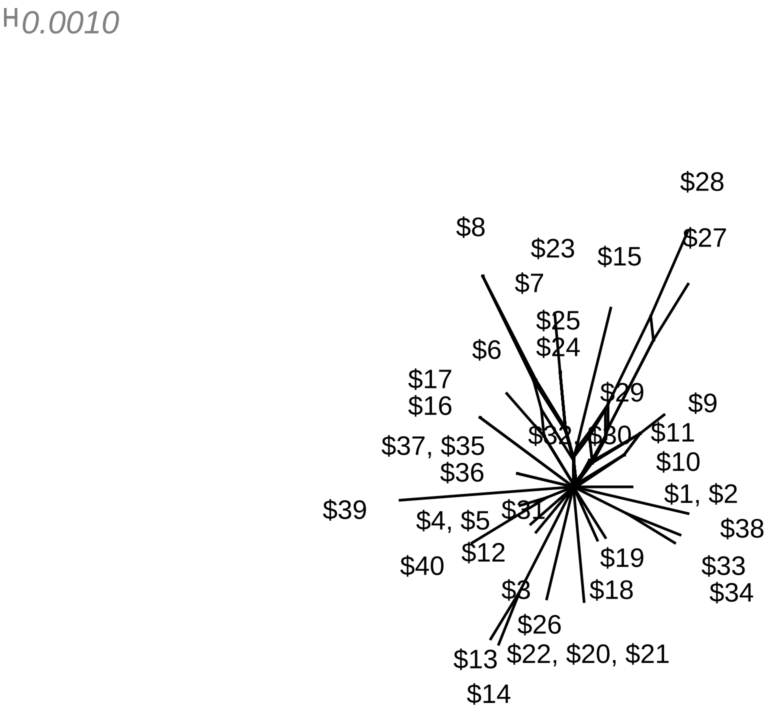
0.0010
$28
$8
$23
$15
$27
$7
$25
$24
$6
$17
$16
$29
$9
$32, $30
$11
$37, $35
$10
$36
$1, $2
$39
$31
$4, $5
$38
$12
$19
$40
$33
$3
$18
$34
$26
$22, $20, $21
$13
$14
